# Evaluation of the relationship between glaucomatous disc subtypes and occurrence of disc hemorrhage and glaucoma progression in open angle glaucoma

**DOI:** 10.1038/s41598-020-77932-z

**Published:** 2020-12-03

**Authors:** Akiko Yamagami, Astuo Tomidokoro, Shun Matsumoto, Yoshio Yamazaki, Keiji Yoshikawa, Junkichi Yamagami, Goji Tomita, Makoto Araie

**Affiliations:** 1 Inouye Eye Hospital, 4-3 KandaSurugadai, Chiyoda-ku, Tokyo 101-0062 Japan; 2 Higashinakano Tomidokoro Eye Clinic, Tokyo, Japan; 3 Ideta Eye Hospital, Kumamoto, Japan; 4 Department of Ophthalmology, Tokai University of Tokyo Hospital, Tokyo, Japan; 5 Yoshikawa Eye Clinic, Tokyo, Japan; 6 Department of Ophthalmology, JR Tokyo General Hospital, Tokyo, Japan; 7 Department of Ophthalmology, Toho University Ohashi Medical Center, Tokyo, Japan; 8 Kanto Central Hospital of The Mutual Aid Association of Public School Teachers, Tokyo, Japan

**Keywords:** Diseases, Medical research

## Abstract

To compare the occurrence of disc hemorrhages (DH) and glaucoma progression in open-angle glaucoma (OAG) patients with different glaucomatous disc types. Prospective, hospital-based, observational cohort study. OAG patients examined between 2000 and 2005, whose discs were classified as typical myopic glaucomatous (MG), generalized enlargement of cup (GE), or focal glaucomatous (FG) disc type were included and followed for 5 years. The first occurrence of DH during follow-up was analyzed using Kaplan–Meier analysis and difference in DH occurrence based on glaucomatous disc type using the Cox proportional-hazards model to adjust for effects of confounding factors. For inter-group comparison of glaucoma progression, the change rate of the mean deviation, Collaborative Initial Glaucoma Treatment Study scores, and fundus photographs were used. Thirty-nine patients with MG-, 18 with FG-, and 17 with GE-disc types were included. No significant inter-group difference was seen in the rate of glaucoma progression. The five-year probability of DH occurrence was much lower with MG- than with FG- or GE-disc types (*P* < 0.0220). The central corneal thickness (*P* = 0.0024) and mean intraocular pressure and its variations (*P* = 0.0450, 0.0219) contributed to DH occurrence. The MG-disc type demonstrated a much lower DH occurrence during follow-up than other disc types.

## Introduction

Disc hemorrhage (DH) is a well-known risk factor for progression of visual field (VF) defects in open-angle glaucoma (OAG)^1–4^, but the mechanism of development of DHs has not been clarified. However, because DHs are most frequently associated with OAG with normal intraocular pressure (IOP) (normal tension glaucoma, NTG)^1,5^, and the occurrence is unaffected even when IOP is reduced with non-surgical treatments^6^, DH may be related to factors other than IOP-dependent damaging processes.

In Eastern Asian countries including Japan^7,8^, the prevalence of myopia is high. Myopia is frequently characterized by a tilted oval disc with a temporal crescent of peripapillary atrophy (PPA) and is a well-known risk factor for OAG^9,10^, which has been suggested to be characterized by structural vulnerability of the myopic disc^11,12^.

Conversely, different distinct appearances of glaucomatous optic discs have been described: a generalized enlargement (GE) of the optic disc cup, focal glaucomatous (FG) optic disc, myopic glaucomatous (MG) optic disc, and senile sclerotic (SS) optic disc; all have different clinical associations and/or prognoses^13–17^. Probably because of the high prevalence of high myopia^8^ and OAG^18^ in Japan, the MG disc is seen relatively frequently in outpatient clinics^19^. Thus, the relationship between the structural changes of the optic disc caused by myopia^11,12^ as a risk factor for OAG^9,10^, and the occurrence of DH as an important risk factor for glaucoma progression^1–3^, is an interesting clinical issue. To the best of our knowledge, however, this issue has not been addressed yet. We report the results of a 5-year prospective comparative observational cohort study of the occurrence of DH and the rate of glaucoma progression in eyes with OAG with different types of glaucomatous optic discs.

## Methods

### Inclusion criteria

This study included subjects who were recruited from 2035 POAG patients with elevated or normal IOP, were examined at one of the following institutions between 2000 and 2005: Ophthalmology Department, University of Tokyo Hospital; Ophthalmology Department, Tokyo Teishin Hospital; Ophthalmology Department, Nihon University School of Medicine, Itabashi Hospital; Yoshikawa Eye Clinic; and Ophthalmology Department, Japan Railway Company Tokyo General Hospital. The institutional review board of each hospital approved the study, which adhered to the tenets of the Declaration of Helsinki. Patients who were included agreed to participate in the study, provided written informed consent, and met the following criteria: age less than 65 years; no clinically relevant cataract that would hamper visual field (VF) evaluation over the next 5 years; no ocular diseases other than glaucoma; no systemic hypertension or diabetes; no use of antihypertensive drugs or calcium antagonists that may affect disease progression; two or more previous VF tests performed with the Humphrey Field Analyzer (HFA) central 30-2 (HFA30-2) full-threshold or Swedish Interactive Threshold Algorithm-Standard (SITA-S) test program (Carl Zeiss Meditec, San Leandro, CA) with reliable (fixation loss, false positive and negative rates < 33% for the former and fixation loss < 20%, and false positive < 15% for the latter) and reproducible results that met the Anderson and Patella^20^ criteria for the diagnosis of glaucomatous VF defects; typical glaucomatous changes in the optic disc or peripapillary retina that were compatible with the VF test results based on the hemifields, such as a vertical cup-to-disc ratio of ≥ 0.7, 0.1 or less rim/disc diameter ratio in the superior or inferior portion of the optic disc (narrowing of the rim or notch), or retinal nerve fiber bundle defects; bilateral mean deviation (MD) >  − 15.0 decibels (dB); and bilateral refractive error with a spherical equivalent ≥  − 10.0 D. One eye of each patient was examined and the eye with the better MD was selected if both eyes met the inclusion criteria. Eyes with pathologic myopia or those with anomalous discs such as tilted disc syndrome were carefully excluded. Criteria for diagnosing pathologic myopia were the presence of posterior staphyloma, localized and/or diffuse chorioretinal atrophy, ridge at temporal side of the disc, and/or peripapillary findings consistent with that later reported as intrachoroidal cavitation^21,22^; the criteria for diagnosing tilted disc were a disc with a minimum to maximum disc diameter ratio less than 0.75, conus or crescent constricted to the inferior to inferonasal region, posterior staphyloma and/or atrophy of the pigment epithelium in the inferonasal fundus, and situs inversus^23,24^.

### Selected cases

The disc photographs of the eyes of 230 OAG patients who fulfilled the inclusion criteria were screened to identify patients with glaucomatous disc subtypes, that were considered typical MG, GE, FG, or SS discs.

Seventy-four eyes of 74 patients with OAG were followed prospectively, i.e. 39 eyes of 39 patients with MG discs, 17 eyes of 17 patients with GE discs, and 18 eyes of 18 patients with FG discs. Among the included eyes, 80% had untreated IOP within normal range (NTG).

### Follow-up of cases

**Figure 1:**
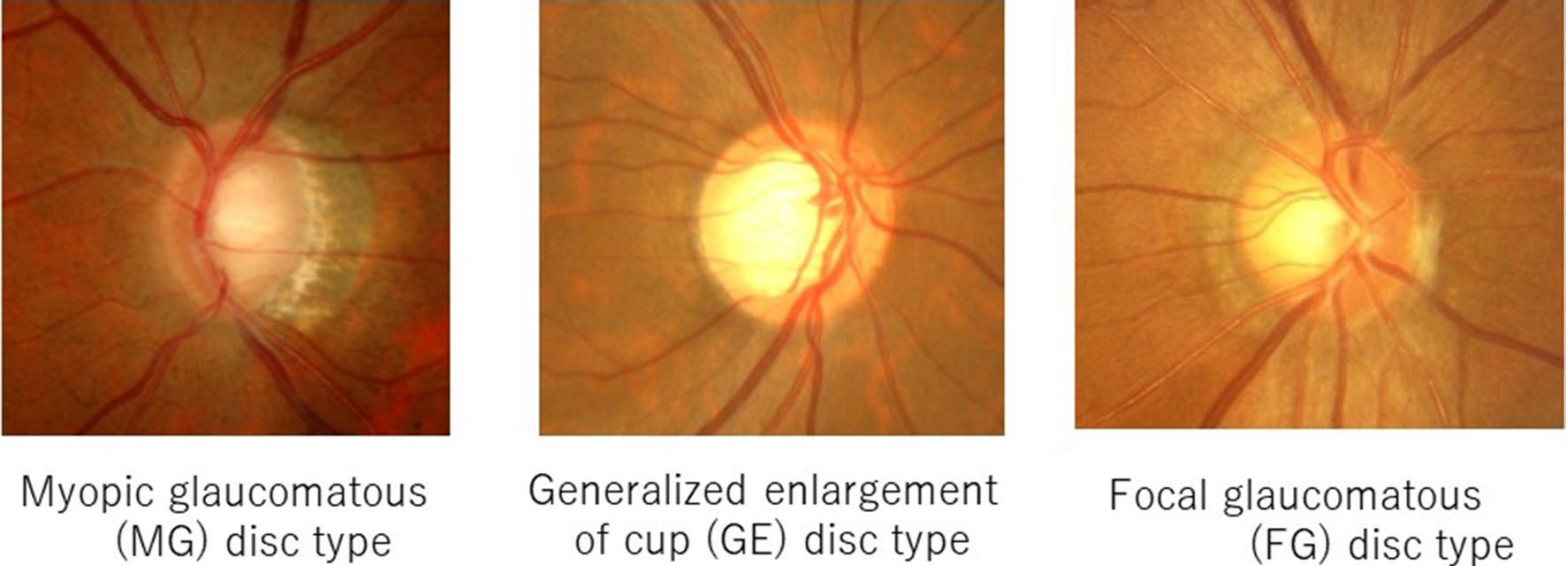
Photographs of representative glaucomatous optic disc types. Photographs of representative myopic glaucomatous (MG) disc type, generalized enlargement of cup (GE) disc type and focal glaucomatous (FG) disc types are seen.

Four glaucoma specialists independently assessed the appearance of the optic discs based on current standards^15^ using non-stereo optic disc photographs with a 45° viewing angle, and classified the cases as having typical MG, GE, FG, or SS discs in at least one eye. Only eyes where diagnostic agreement of the four investigators was reached were included. As a preliminary study, a separate group of disc photographs of 50 eyes of 50 OAG patients were independently classified into the five categories (MG, GE, FG, or SS and others, including all optic discs not considered to have typical MG, GE, FG, or SS discs) twice, at intervals ranging from 7 to 14 days. Classification of the glaucomatous disc types showed modest agreement among the four investigators (Fleiss κ = 0.47 [95% confidence interval: 0.41–0.55]) at both first and second sessions, and intra-individual reproducibility was good (Cohen κ = 0.65 [0.48–0.82]–0.84 [0.72–0.96]). If the glaucomatous optic disc subtype of an eye was determined by complete consensus of the four investigators, the reproducibility of the classification of the glaucomatous optic disc subtype was excellent (Cohen κ = 0.92 [0.83–1.00]). Since only two eyes were determined to have SS discs under the current conditions, the eyes with MG, GE, or FG discs were compared. Photographs of representative optic discs of each type are shown (Fig. 1).

Regarding glaucoma treatment, only untreated patients or those treated with either latanoprost, timolol, or carbonic anhydrase inhibitor eye drops, or a combination of those drugs were included. The observation period was 5 years; IOP was measured using applanation tonometry, and the examination for the incidence of DHs generally was performed every 2 months. Each patient’s VF was examined every 6 months using the same program (HFA 30-2 full-threshold or SITA-S test program); in addition, fundus photographs with a 30° angle of view were acquired every year.

**Figure 2:**
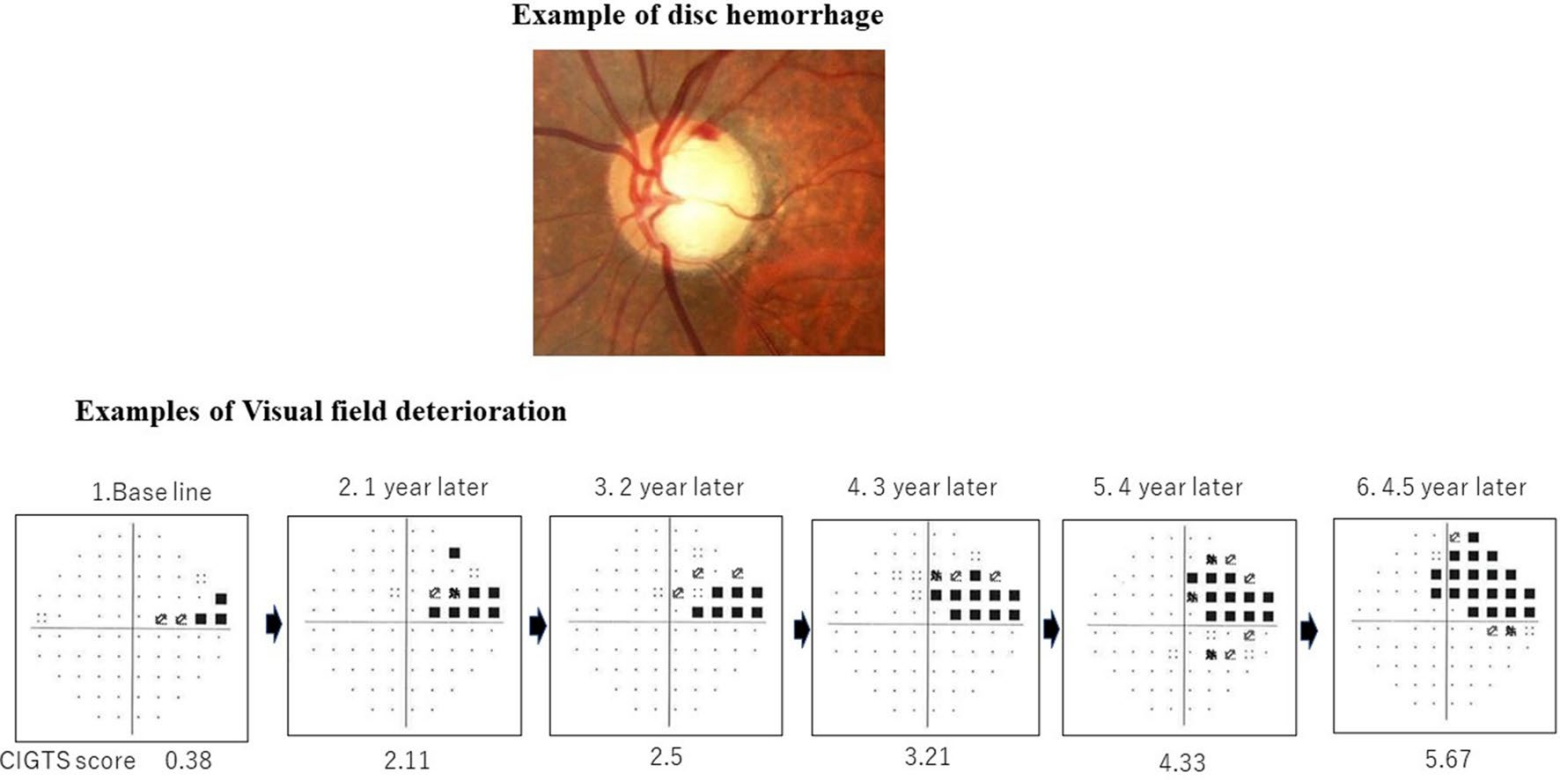
An example of disc hemorrhage and visual field series, which showed deterioration. Flamed-shaped bleeding was seen in the optic disc tissue crossing the disc margin. Visual field was considered to have progressed when the Collaborative Initial Glaucoma Treatment Study (CIGTS) score increased by three points or more compared with the baseline^31^. Each visual field test result was obtained at an interval of 1 year using HFA 30–2 full-threshold program. Judged to have progressed VF No.5.

Six glaucoma subspecialists examined the patients for DH using direct ophthalmoscopy with pupillary dilation when necessary, and splinter or flamed-shaped bleeding in the optic disc tissue crossing the disc margin were categorized as glaucomatous DH (Fig. 2)^1,5^. A preliminary study with another group that included 50 eyes of 50 OAG patients, who were examined by the six investigators who followed up patients and determined the presence or absence of glaucomatous DH in the main study, showed excellent agreement (Fleiss κ = 0.93 [0.88–0.98]) regarding the presence or absence of glaucomatous DH. The HFA 30-2 full-threshold or SITA-S test program was used for VF testing, and each patient was examined with the same program during follow-up visits. The central corneal thickness (CCT) was measured using ultrasonography or noncontact specular microscopy. The results of the specular microscopy measurements were converted to ultrasonography equivalents using a conversion formula (Y = 57.2 + 0.93X where X = the ultrasonography results and Y = specular microscopy results)^25^.

When the first occurrence of DH was observed during follow-up (the 1st DH during follow-up), it was defined as an event in the life-table method. When the investigator was unsure if the DH was glaucomatous, a fundus photograph was obtained for later evaluation, discussion, and consensus among the six investigators who followed the patients. Patients were excluded from the life-table analysis during the observation period and treated as censored cases if they used antiglaucoma drugs other than those mentioned previously, underwent intraocular surgery, their IOP increased to 25 mmHg or higher or their MD decreased to − 15 dB or lower with reproducibility at least in one eye, or started the use of oral drugs for the treatment of systemic hypertension or diabetes.

### Method of analysis of occurrence of DH

The 1st DH during follow up, defined as an event in the life-table method in each glaucomatous disc subtype group was analyzed using the Kaplan–Meier life-table method. The relationship between the 1st DH during follow up and the glaucomatous disc subtypes was adjusted for age, average IOP during the 5-year follow-up period and its standard deviation (SD) or that divided by the mean (coefficient of variation [CV] values for IOP), baseline mean deviation (MD) value, CCT, and vertical cup/disc ratio using the Cox proportional-hazards model, while treating these factors and the glaucomatous disc subtypes as explanatory variables. The spherical equivalent of refractive errors and β-peripapillary (PPA)/disc area ratio were not included because of the apparent high correlation between these parameters and the MG discs. IBM SPSS Statistics version 20 (IBM Japan. Inc., Tokyo) was used to analyze the data.

The prevalence of each glaucomatous disc subtype in Japan is unknown. However, roughly half of the glaucomatous disc subtypes in eyes with POAG seen in clinical practice in Japan are MG discs^19^. The number of patients to be enrolled in the present study was calculated such that a difference of 33% or greater in the occurrence of DH between the MG disc group and non-MG disc groups (GE, FG, and SS discs combined) could be detected with an α of 0.05 and (1 − β) of 0.8, assuming that the overall occurrence of DH during the 5-year follow-up period was 50%^5,26,27^. The target number of eyes with MG discs and those with non-MG discs was 36 each^28^.

### Method of analysis of glaucoma progression

The VF results of each group were compared based on the change rate of MD using a linear mixed model^29^, assuming there was no significant difference in the MDs between the HFA 30-2 full-threshold and SITA-S test programs^30^. Further, VF damage was considered to have progressed when the average Collaborative Initial Glaucoma Treatment Study (CIGTS) score increased by three points or more compared with the baseline score^31^; the average of 2 evaluations performed within 3 months of enrolment in the study, at two consecutive visits (Fig. 2) were considered. Two investigators who were masked to the clinical information analyzed the VF test results. The three members of the fundus photograph evaluation subgroup assessed the optic disc appearance and retinal nerve fiber layer changes independently under masked conditions, based on each pair of baseline and follow-up photographs obtained every year. Each member recorded the differences observed between the two photographs. Progression was flagged in cases in which all three investigators determined that the worse photograph was the follow-up one. If the investigators did not reach agreement, consensus was reached by discussion.

## Results

### Patients’ demographic data

Statistical analysis showed significant intergroup differences in the spherical equivalent of refractive errors, baseline MD value, vertical cup/disc ratio, and β-PPA/disc area ratio. No significant intergroup differences were seen in the male/female ratio, age, blood pressure (systolic and diastolic), (OAG with normal IOP or NTG)/(POAG with elevated IOP) ratio, maximal IOP recorded before enrollment, baseline IOP, CCT, and medication (Table 1).

**Table 1:** Patient demographic data.

	MG group	FG group	GE group	*P* Value
Number of eyes (Patients)	39 (39)	18 (18)	17 (17)	
Male/female	23/16	9/9	6/11	0.2625*
Age (years)	48.7 ± 8.4	49.9 ± 10.4	54.3 ± 9.3	0.1144^†^
Blood pressure (systolic)	121.8 ± 16.3	124.4 ± 15.3	126.5 ± 10.7	0.5389^†^
(diastolic)	79.0 ± 11.6	78.5 ± 9.2	79.2 ± 9.3	0.8505^†^
NTG/POAG	33/6	13/5	12/5	0.3855*
Refractive error (diopters)	-6.0 ± 2.3	-2.0 ± 3.0	-1.6 ± 2.4	< 0.001^†^
Baseline IOP (mmHg)	13.1 ± 1.9	13.6 ± 1.6	13.2 ± 1.7	0.7648^†^
Maximum IOP (mmHg)	18.0 ± 2.9	18.1 ± 2.8	18.8 ± 3.2	0.6195^†^
Baseline MD (dB)	-5.3 ± 3.1	-3.0 ± 2.2	-7.1 ± 2.8	< 0.001^†^
Baseline CIGTS scores	4.23 ± 3.34	2.10 ± 2.07	5.03 ± 2.73	0.064^†^
Central corneal thickness (μm)	542.7 ± 33.1	529.3 ± 31.0	521.6 ± 22.9	0.3499^†^
Vertical cup/disc ratio	0.68 ± 0.09	0.70 ± 0.07	0.80 ± 0.08	< 0.001^†^
β-PPA/disc area ratio	0.90 ± 0.77	0.15 ± 0.23	0.15 ± 0.23	< 0.001^†^
**Topical medication (eyes)**				
No medication	9/39	3/18	2/17	0.6899*
Latanoprost	24/39	13/18	14/17	0.7979*
Timolol	11/39	12/18	8/17	0.2213*
Carbonic anhydrase inhibitor	4/39	4/18	2/17	0.5659*

MG, myopic glaucomatous; FG, focal glaucomatous; GE, generalized enlargement of optic disc cup; NTG, normal tension glaucoma; dB, decibels; POAG, primary open-angle glaucoma; IOP, intraocular pressure; MD, mean deviation; β-PPA, β-peripapillary atrophy; CIGTS, Collaborative Initial Glaucoma Treatment Study; NS, not significant.

*Chi-square test.

^†^Kruskal–Wallis test.

The average achievement rate of the desired follow-up were 95.9 ± 7.3, 95.2 ± 7.6 and 96.3 ± 6.3% for the MG, FG, and GE groups, respectively. The times between visits and mean IOP values during follow-up were 1.8 ± 0.7, 2.0 ± 0.6, and 1.7 ± 0.5 months and 13.1 ± 1.9, 13.6 ± 1.6, 13.3 ± 1.7 mmHg for the MG, FG, and GE groups, respectively. No intergroup differences were observed in any of these parameters (*P* = 0.8996, *P* = 0.3433, *P* = 0.6291, One-way analysis of variance).

### Censored cases

Seven patients in the MG group (one who had a hospital transfer, one who underwent buckling procedure for retinal detachment, one who underwent cataract surgery due to acute cataract development, and four who withdrew consent), three patients in the FG group (two who started drugs for systemic hypertension and one who was using a topical antiglaucoma drug apart from those specified in the Methods), and two patients in the GE group (one who withdrew consent and one who underwent laser trabeculoplasty due to insufficient IOP control) withdrew during the 5-year follow-up period and were treated as censored cases in the Kaplan–Meier analysis.

### Cumulative probabilities of the 1st DH during follow up

**Figure 3:**
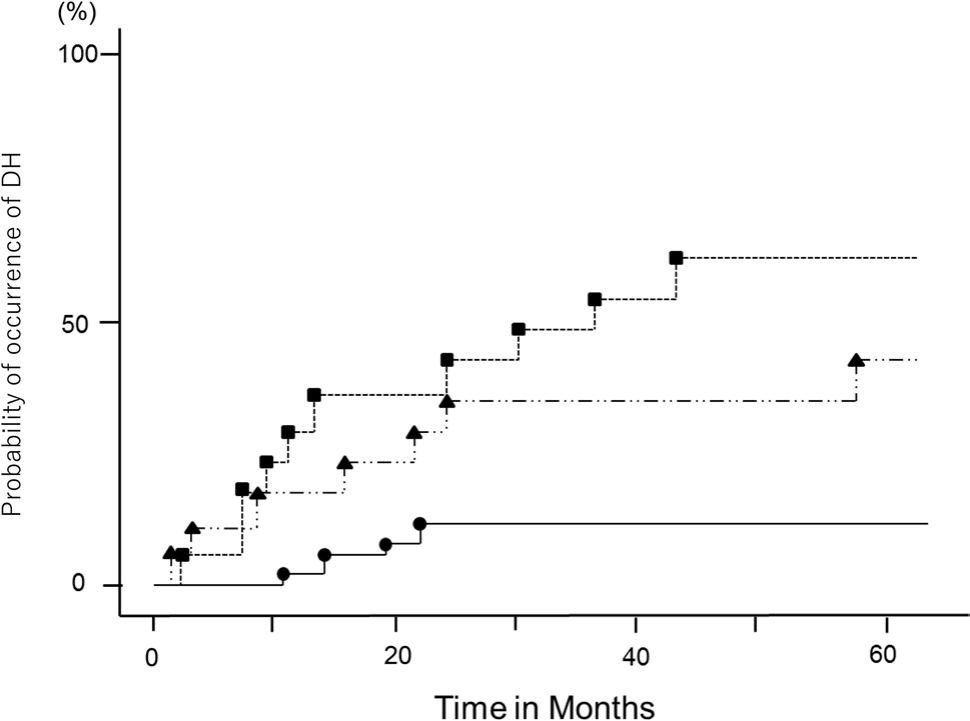
Cumulative probabilities of the occurrence of disc hemorrhages (DH) (Kaplan–Meier method). The probability values at 5 years are 10.4% ± 4.9% (standard error), 40.7% ± 12.1%, and 60.3% ± 12.2% for the myopic glaucomatous (MG), focal glaucomatous (FG), and generalized enlargement of cup (GE) disc types, respectively (MG vs. FG disc type, *P* = 0.0032; MG vs. GE disc type, *P* = 0.0003; FG vs. GE disc type, *P* = 0.8441; log-rank test). The closed circles, triangles, and squares indicate that the MG, FG, and GE disc types, respectively.

The cumulative probabilities of t the 1st DH during follow-up as shown by Kaplan–Meier analysis were 10.4% ± 4.9% (standard error) in the MG group, 40.7% ± 12.1% in the FG group, and 60.3% ± 12.2% in the GE group at 5 years, respectively (MG vs. FG group, *P* = 0.0032, MG vs. GE group, *P* = 0.0003; FG vs. GE group, *P* = 0.8441; log-rank test; Fig. 3).

### Results of Cox proportional-hazards model analysis

The Cox proportional-hazards model analysis showed significant contributions of the glaucomatous disc subtype (*P* = 0.0077), CCT (*P* = 0.0024), mean of the IOP recorded during follow-up (*P* = 0.0450), and SD of IOP recorded during follow-up (*P* = 0.0219) to the 1st DH during follow up (Table 2). In the analysis performed by converting SD to CV, we found that glaucomatous disc subtype (*P* = 0.0087), CCT (*P* = 0.0020), and CV of IOP recorded during follow-up (*P* = 0.0173) significantly contributed to the 1st DH during follow up.

**Table 2:** Results of cox proportional-hazards model analysis.

Variable	HR (95% CI)	*P* Value
Disc appearance		0.0077
MG vs. FG group	5.07 (1.26–25.00)	0.0220
MG vs. GE group	6.82 (1.78–30.53)	0.0048
FG vs. GE group	1.34 (0.36–4.89)	0.6559
Age	1.02 (0.96–1.08)	0.5501
Central corneal thickness	0.97 (0.96–0.99)	0.0024
Baseline mean deviation value	0.95 (0.78–1.16)	0.6135
Vertical cup/disc ratio	0.07 (6.6e-5–88.54)	0.4641
Mean IOP during follow-up	1.34 (1.01–1.79)	0.0450*
SD of IOP during follow-up	0.13 (0.02–0.76)	0.0219*

MG, myopic glaucomatous; FG, focal glaucomatous; GE, generalized enlargement of optic cup disc; IOP, intraocular pressure; SD, standard deviation; CI, confidence interval; HR, hazard ratio.

*Not selected as a significantly contributing factor or not used when coefficient variation (CV) of IOP during follow-up was used as an explanatory variable instead of the SD of IOP during follow-up.

### Cumulative probability of glaucoma progression

The rates of MD changes during the 5-year period were − 0.02 ± 0.08, − 0.07 ± 0.11, and − 0.15 ± 0.11 dB/year in the MG, FG, and GE groups, respectively, with no significant intergroup differences (MG vs. FG group, *P* = 0.7148, MG vs. GE group, *P* = 0.3540; FG vs. GE group, *P* = 0.6159; linear mixed model). Decreases in the CIGTS scores during follow-up were seen in six, two, and two patients in the MG, FG, and GE groups, respectively; six patients in the MG group did not present with DH, whereas two patients each in the FG and GE groups presented with DH. Fundus photograph-based progression was seen in six (one showed DHs and five did not show any DHs), three (one showed DH and two did not show any DHs), and six (four showed DHs and two did not) cases in the MG, FG, and GE groups, respectively. One patient each in the MG and GE groups also had showed progression in both, fundus photographs and CIGTS scores. The probabilities of disease progression based on Kaplan–Meier analysis and defined by CIGTS score deterioration were 19.4%% ± 7.2% (standard error), 15.2% ± 10.0%, and 14.3% ± 9.4% in the MG, FG, and GE groups, respectively, with no significant intergroup differences (MG vs. FG group, *P* = 0.7624, MG vs. GE group, *P* = 0.7541; FG vs. GE group, *P* = 0.9796; log-rank test).

**Figure 4:**
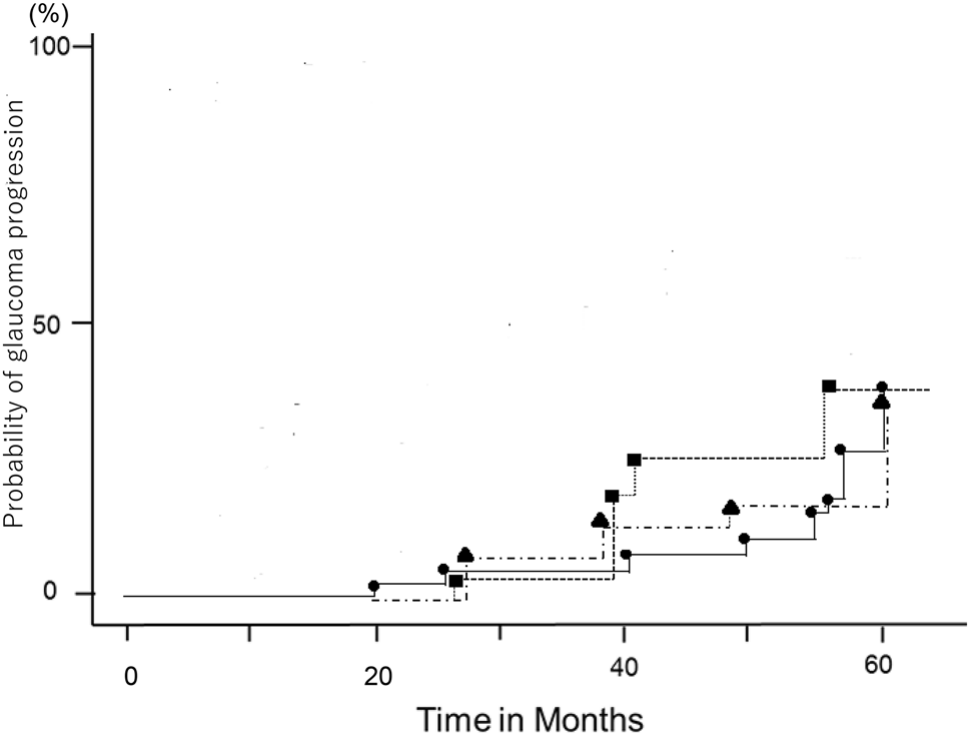
Probabilities of glaucoma progression defined by CIGTS score deterioration and/or fundus photograph deterioration. The probabilities of glaucoma progression defined by CIGTS score deterioration and/or fundus photograph deterioration were 39.8% ± 10.1%, 40.0% ± 14.0%, and 42.9% ± 13.2% for the myopic glaucomatous (MG) disc type, focal glaucomatous (FG) disc type, and generalized enlargement of cup (GE) disc type, respectively, with no significant intergroup differences (MG vs. FG disc type, *P* = 0.7652; MG vs. GE disc type, *P* = 0.4831; FG vs. GE disc type, *P* = 0.6228; log-rank test). The closed circles, triangles, and squares indicate the MG, FG, and GE disc type, respectively.

The probabilities of glaucoma progression defined by CIGTS score deterioration and/or fundus photograph deterioration were 39.8% ± 10.1% (standard error), 40.0% ± 14.0%, and 42.9% ± 13.2% in the MG, FG, and GE groups, respectively, with no significant intergroup differences (MG vs. FG group, *P* = 0.7652; MG vs. GE group, *P* = 0.4831; FG vs. GE group, *P* = 0.6228; log-rank test; Fig. 4).

However, the numbers of eyes with DHs that exhibited progression were 1 (10%) of 11, 3 (60%) of 5, and 4 (66%) of 7 in the MG, FG, and GE groups, respectively, with a significant intergroup difference (*P* = 0.0189; Fisher’s exact method).

## Discussion

The aim of this study was to compare the occurrence of DHs and glaucoma progression in eyes with OAG, based on different glaucomatous disc subtypes. Differences in clinical associations and prognoses associated with the appearances of glaucomatous optic discs have been reported; the FG disc type is more likely to be associated with the female sex, local VF defects, migraine and vasospasm, and a faster rate of progression^13,15,17^; the GE disc type is more likely to be associated with higher untreated IOP and diffuse VF defects^13,15^; and the SS disc type is associated with older age, systemic cardiovascular diseases, compromised retrobulbar circulation, thinner peripapillary choroid, and a slower rate of progression^13–16^. The MG disc type is more frequently found in Asians and is associated with myopia^13,15^. However, to the best of our knowledge, no longitudinal studies have compared the occurrence of DH during follow-up among eyes with different glaucomatous optic disc appearances.

According to the criteria employed for the present study, 76 out of 230 eyes (230 patients) were judged to have typical MG, FG, EG, or SS disc type. For the four investigators who independently classified the disc subtypes of Japanese OAG patients, inter- and intra-observer agreement κ values and the ratio of eyes classified based on the complete consensus of the observers (76/230 = 33%) were similar to those reported by Nicolela et al. (κ values of 0.40, 0.51–0.85, respectively, and 35%)^32^. Inclusion of subject eyes that may be recognized as atypical MG, FG or GE disc type may have confounded the analysis. The cumulative probability of the 1^st^ DH during follow up in the study subjects was 10.0% ± 4.9% for the MG disc type; this was significantly (*P* < 0.0032–0.0003; log-rank test) lower than the values of 40.7% ± 12.1% and 60.3% ± 12.2% recorded in the FG and GE disc types, respectively. The occurrence rates of DHs in eyes with the FG or GE disc type did not differ substantially from the reported incidence rates in eyes with POAG and normal IOP during follow-up^5,30^. The Cox proportional-hazards model also showed that glaucomatous disc types contributed significantly to the 1^st^ DH during follow-up after adjustment for other confounding factors described below. It also showed that a thinner CCT, mean IOP, and greater fluctuation of IOP (SD of IOP or CV of IOP) during follow-up were associated with a higher occurrence of DHs. Since DHs are generally associated with glaucoma progression^1–3^ and a thinner CCT^33,34^, higher IOP, or greater IOP fluctuation in glaucomatous eyes with a low mean IOP^35^ are also reportedly risk factors for glaucoma progression; association of these parameters with the occurrence of DH seems to be compatible with the information in the available literature. The rate of DH recurrence was 0% in eyes with the MG subtype, 62.5% in eyes with the FG subtype, and 44.4% in eyes with the GE subtype; this is consistent with the lower occurrence of DH in the MG subtype. Despite the much lower occurrence or recurrence of DH in the MG group, glaucoma progression defined by the rate of MD change or deterioration of CIGTS scores and/or fundus photographic findings appeared very similar among the MG, FG, and GE disc type, and the association of DH occurrence with glaucoma progression was evident in the FG and GE disc type but not in the MG disc type. Myopia is a risk factor for OAG^9,10^, which is presumed to be related to structural vulnerability of the disc to glaucoma-causing insults that occur due to myopic changes^11,12^. However, Sohn et al.^36^ reported that myopia is not always a risk factor for progression in treated NTG; this agrees with the finding in the present study in that the disease progression rate was similar among eyes with the MG, FG, and GE disc type within the same IOP range.

The pathogenesis of DH is not clearly understood. Findings reported in the available literature have suggested underlying IOP-independent vascular or circulatory disorders^5,37^; however, the fact that sufficient IOP reduction decreased the occurrence of DH suggested the involvement of an IOP-dependent mechanical insult^29,30^. Previous studies that utilized laser Doppler flowmetry or colour Doppler imaging reported reduced choroidal or peripapillary blood flow or central retinal artery blood velocity in myopic eyes^38,39^. Collectively, the results of the present study show that the MG disc type is associated with a much lower occurrence of DH during follow-up, but with a similar rate of disease progression as the FG and GE disc types; IOP levels of approximately 13.5 mmHg may suggest that there is a difference in the contribution of glaucoma-related damaging factors, possibly relating to myopic structural change^11,12^.

The present study had several limitations. Firstly, only a few eyes showed typical SS disc type on examination during patient recruitment; this may have resulted partly from the inclusion criteria, according to which all subjects were younger than 65 years and did not have systemic hypertension. Further, a less characteristic appearance of the SS disc type may be at least partly responsible for the difficulty in reaching unanimous agreement when determining the glaucomatous disc appearances. Future studies on the relationship between the SS disc type and DH occurrence are needed. Secondly, the number of eyes included in the present study was not large enough. The sample size, however, allowed for the detection of a 33% difference in the 1st DH during the 5-year follow-up with reasonable power of detection and an α error, and the statistically significant difference detected between the MG and FG or GE groups was 30%–50%, which would guarantee the robustness of the difference currently observed in the occurrence of DH. Thirdly, DH was not detected by evaluation of stereo disc photographs, but by direct ophthalmoscopy through dilated pupils. Budenz et al.^40^ reported that DH could be detected better on stereo disc photographs than by clinical examinations. However, one study reported an acceptable agreement between clinical examinations and evaluation with stereo disc photographs for detecting DHs^41^. Bengtsson et al.^6^ also reported similar results when clinical examinations were performed at intervals of 3 months. In the present prospective study, the glaucoma experts evaluated optic discs by direct ophthalmoscopy (through dilated pupils if needed) every 2 months, and inter-investigator agreement regarding the presence or absence of DHs was excellent. Thus, DH detection in this study may not have been as good as that performed by evaluating stereo disc photographs taken every 2 months; however, regarding the inter-group comparison of the presence or absence of DH using the same method, the current conclusion was minimally affected by the method of DH detection. In almost half the patients, VF tests were performed using the full-threshold program throughout the follow-up period, whereas the other half was tested using the SITA-S program throughout the follow-up period. Thus, the calculated change rate of the MD, which was relatively small (about − 0.1 dB/year), may not be accurate enough. However, this value is in accordance with the results from a study that included approximately 170 Japanese patients with NTG and undertreated IOP of approximately 13 mmHg, who were prospectively followed using the same VF test program (full-threshold program)^42^; the mean IOP value in the aforementioned study is similar to the mean IOP of approximately 13.5 mmHg recorded during the follow-up of the study subjects in the present study. Further, considering that the mean IOP during follow-up was 13.5 mmHg, the rate of progression was in line with that recorded in previous prospective studies^43,44^. A similar rate of glaucoma progression in eyes with the MG, FG, and GE disc type also suggested that it is unlikely that non-glaucomatous myopic eyes were misdiagnosed as having myopic glaucoma and included in the MG disc type group in the present study. The number of the subjects in our study was not enough to detect a small difference in the rate of glaucoma progression. However, the difference observed in the 1^st^ DH during follow up between the MG and FG or GE subtypes (10% vs. 40%–60% with standard errors of 5%–12%) during the 5-year follow-up period was evidently different from the difference observed in the rate of glaucoma progression (40% vs. 40%–43% with standard errors of 10%–14%). Finally, it should be noted that the results of the present study are to be applied to eyes with an MG disc type and not to those with myopia, since an eye with myopia does not always have a typical MG disc type.

In summary, we prospectively followed OAG eyes with MG, FG, and GE disc types for 5 years and compared the occurrence of DHs in these eyes. Although no differences in glaucoma progression and IOP were observed among the three groups, eyes with an MG disc type had a substantially lower occurrence of DH during follow-up. This finding suggests that the mechanism of glaucoma progression may not be the same among the three distinct glaucomatous disc types; this may be related partly to characteristic structural changes in the MG disc type.

## Data Availability

As mentioned in the text, the study participants were recruited between 2000 and 2005 for this 5-year longitudinal observational study, and we believe that almost all of the journals dealing with clinical studies did not require the authors’ disclosure of data availability to the third party in between 2000 and 2005. Accordingly, the informed consent acquisition document for this study did not include a sentence asking whether a study participant agreed to the sharing of his/her personal information, i.e. follow-up results, with a third party such as an editorial office of a scientific journal. The study participants agreed to participate in the study under the understanding that the data obtained during the follow-up period were immediately anonymized, strictly controlled by the investigators and institute, available only to the investigators listed in the informed consent acquisition document, and only used to study the occurrence of disc haemorrhage during the follow-up period, with the results reported in authorized academic meetings and published in authorized scientific journals. Therefore, the data will be theoretically available to a third party if the authors can successfully contact all study participants, who completed their follow-up in 2011, and obtain written informed consent for sharing of their data with a third party.
